# The Annual Address before the Hom. Med. Society of N. Y.

**Published:** 1884-07

**Authors:** 


### BOOK NOTICES.

Annual Address Before the Homœopathic Medical Society of the State of New York, February 12th, 1884. By Everitt Hasbrouck, M. D., the President.

In an address of more than the usual ability, Dr Hasbrouck considers the question: Are we justified in maintaining a distinctive position? And his conclusion is that "until general acknowledgment shall have been secured, we are justified in maintaining a name and position in keeping with the truth we have espoused."

